# Fatal Sickness among the Otoe Indians

**Published:** 1855-01

**Authors:** 


Fatal Sickness among the Otoe Indians.—Accounts from the villages of the above tribe go to show that they are dying in great numbers. It is supposed that the pork or bacon received as annuity, becoming musty or tainted, is the occasion of this mortality. What number has already died we have not been able to learn. We doubt not that the Agent will immediately look into the matter, and give such assistance as is necessary.—*Council Bluff Eagle*, *Oct.* 31, 1854.

